# Faster, Deeper, Better: The Impact of Sniffing Modulation on Bulbar Olfactory Processing

**DOI:** 10.1371/journal.pone.0040927

**Published:** 2012-07-17

**Authors:** Frédéric Esclassan, Emmanuelle Courtiol, Marc Thévenet, Samuel Garcia, Nathalie Buonviso, Philippe Litaudon

**Affiliations:** Centre de Recherche en Neurosciences de Lyon (CRNL) Equipe Olfaction : du codage à la mémoire, CNRS UMR 5292 - INSERM U1028 - Université Lyon 1 – Université de Lyon, Lyon, France; INRA-UPMC, France

## Abstract

A key feature of mammalian olfactory perception is that sensory input is intimately related to respiration. Different authors have considered respiratory dynamics not only as a simple vector for odor molecules but also as an integral part of olfactory perception. Thus, rats adapt their sniffing strategy, both in frequency and flow rate, when performing odor-related tasks. The question of how frequency and flow rate jointly impact the spatio-temporal representation of odor in the olfactory bulb (OB) has not yet been answered. In the present paper, we addressed this question using a simulated nasal airflow protocol on anesthetized rats combined with voltage-sensitive dye imaging (VSDi) of odor-evoked OB glomerular maps. Glomerular responses displayed a tonic component during odor stimulation with a superimposed phasic component phase-locked to the sampling pattern. We showed that a high sniffing frequency (10 Hz) retained the ability to shape OB activity and that the tonic and phasic components of the VSDi responses were dependent on flow rate and inspiration volume, respectively. Both sniffing parameters jointly affected OB responses to odor such that the reduced activity level induced by a frequency increase was compensated by an increased flow rate.

## Introduction

The understanding of sensory information processing is a major challenge for neuroscience, as this processing is the foundation that generates representations of the world in both humans and animals. Numerous works have already clearly established several common features concerning the processing of various sensory modalities. One of the most important features is the cerebral topographic coding of stimuli. This topographic coding consists in the apparition of orderly cortical representations or maps that reflect the stimulation of a specific sensory receptor subgroup [1].

In contrast to the tactile somatotopic, visual retinotopic, and auditory tonotopic maps, the existence of such a topographic coding for olfactory stimuli has been strongly debated [2]–[5]. Although visual, tactile, and auditory stimuli can be easily described and discriminated using a small number of continuous variables, the complexity of olfactory molecules has prevented the establishment of classifications based on a limited set of characteristics. However, an increasing number of studies have highlighted the presence of an olfactory chemotopic coding system in the insect antennal lobe and mammalian olfactory bulb (OB) [6]–[8]. A single odorant activates only a small and specific subgroup of olfactory sensory neurons that converge onto a limited number of glomeruli in the OB [9]. Hence, in the OB, the identity of odorants is associated with a unique spatial map.

In addition to this spatial coding, olfactory information processing is also based upon dynamic mechanisms that depend on the physical method by which stimuli are led to their sensory receptors. In mammals, the respiration cycle itself provides the mechanism for odor stimuli sampling. Even among the very first electrophysiological studies [10], the powerful influence of breathing was observed in the OB and olfactory cortex. Thus, respiratory modulation of both local field potential (LFP) signals and unitary activities has been extensively described in the OB and piriform cortex [11]–[17].

In addition to its role as the vector for odor molecules, respiratory dynamics have been considered as an integral part of the olfactory percept in both animals and humans by various authors [18]–[20]. It has been shown that rats adapt their sniffing strategy, both in frequency and flow rate, when performing odor discrimination and detection tasks [21]. Therefore, the question of how these sampling variations affect olfactory system activity is of growing interest.

In the past few years, several studies have addressed this question at the OB input terminal level [22]–[25], and most of these studies have focused on the impact of sampling frequency on input dynamics. Although Oka et al. [23] also studied the impact of sniffing flow rate, the effects of different sniffing components were analyzed individually, and they failed to analyze the response dynamics. At the OB output level (i.e., mitral cell activity and local field potential recordings), different studies have noted the importance of sniffing frequency [13], [26] and sniffing flow rate [13], [27].

In the present paper, we studied how the two primary components of sniffing behavior, i.e., frequency and flow rate, jointly contribute to the spatio-temporal representation of odors at the OB level. To achieve this goal, we used voltage-sensitive dye imaging (VSDi) combined with an artificial sniffing paradigm in anesthetized rats. Artificial sniffing allowed the independent control of flow rate and frequency within the physiological range [13]. Contrary to most previous optical imaging studies addressing this question at the OB input terminal level, the responses measured with voltage-sensitive dye arise primarily from sources downstream of the olfactory nerve input [28]. This allows possible reshaping of the input activity by local interneurons at the post-synaptic level to be taken into account. This study, to our knowledge, is the first to give an insight of how both sniffing frequency and flow rate modulate both the amplitude and the dynamics of odor evoked VSDi response. Thus, the present study makes the link between data obtained at the OB input [22]–[25] and output [13], [26], [27] level respectively. To strengthen our conclusions, OB LFPs were also recorded in this experiment.

## Results

### Validation of Artificial Sniffing Paradigm

**Figure 1:**
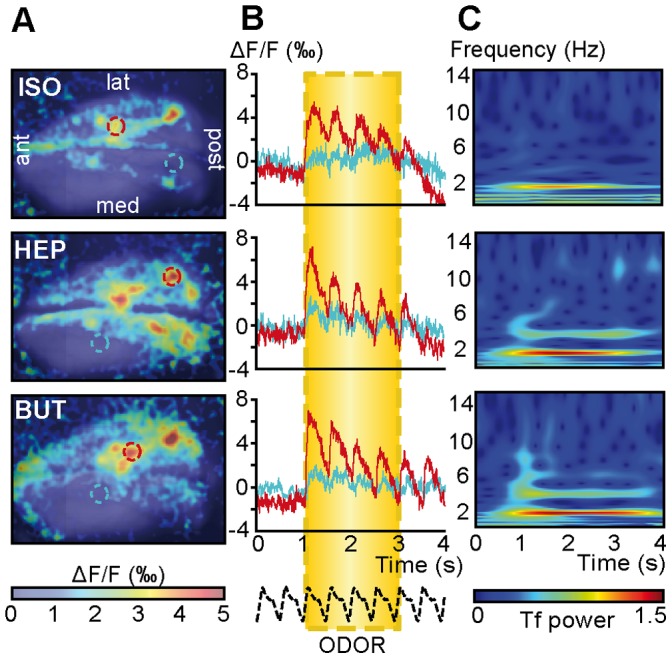
Odor-evoked maps at a resting sniffing frequency. A: VSDi maps evoked by three different odors. Red and blue circles indicate the regions of interest used to compute the optical signal time-course. B: Optical signal time-courses computed from the activated area (red) and non- (low)-activated area (blue). The bottom black dashed line represents the imposed sniffing signal. Yellow box indicates odor stimulation period. C: A time-frequency representation computed from the red signals displayed in B. Time-frequency power (Tf power, arbitrary unit) is color-coded (the color scale is the same for both time-frequency maps).

One concern for using artificial sniffing was the reliability of the sampling parameters. Contrary to most previously used devices [23], [24], our artificial sniffing simulator reproduces both respiratory phases, inspiration and expiration, while reaching high-frequency sniffing (10 Hz) [27] (Fig. S1). Therefore, we first needed to ensure that changes in the response dynamics and/or observed amplitudes at different frequencies were not due to side-effects related to an unstable flow rate between low and high artificial sniffing frequencies. Although it was not possible to measure the absolute flow rate at the entrance of the nostril with our respiratory monitoring setup [29] (see Figure S2 legend), we demonstrated that the actual relative flow rate through the nostril varied linearly with the imposed flow rate whatever the sniffing frequency while matching the imposed frequency (Fig. S1). The second step was to demonstrate that this artificial sniffing protocol combined with VSDi allowed us to record reliable optical responses in the OB. Our goal, herein, was not to analyze the specificity or the reproducibility of odor evoked maps as it has been largely demonstrated in the literature [8], [30]–[32]. We used a 2 Hz artificial breathing frequency that corresponded with the respiratory frequency in anesthetized rats. As previously reported [28], odors evoked specific patterns of post-synaptic activity in the dorsal OB (Fig. 1A), these patterns being similar when using same odor in a given rat. We then analyzed the time-course of the optical responses, focusing on responding glomeruli. As shown in figure 1B, odor presentation evoked a fast increase in fluorescence of approximately 0.6% compared with the basal fluorescence value, which reflected OB activation. As previously reported [28], this response exhibited two components: a tonic component lasting for the duration of odor stimulation and a superimposed phasic component phase-locked with the sampling pattern. Conversely, the non-activated regions did not present a tonic response but were still able to develop a smaller phasic component during odor presentation. Time-frequency maps computed from these responses (Fig. 1C) confirmed that optical signal modulation clearly corresponded with the 2 Hz imposed sniffing frequency.

The following analyses focused on the optical response time-course parameters. As no statistical differences were observed between the odors, data from all odors were pooled for the following analyses. Thus a total of 536 recordings obtained from 20 rats were included in statistical analysis.

### Sniffing Frequency Strongly Impacts the Phasic Component of VSDi Responses

**Figure 2:**
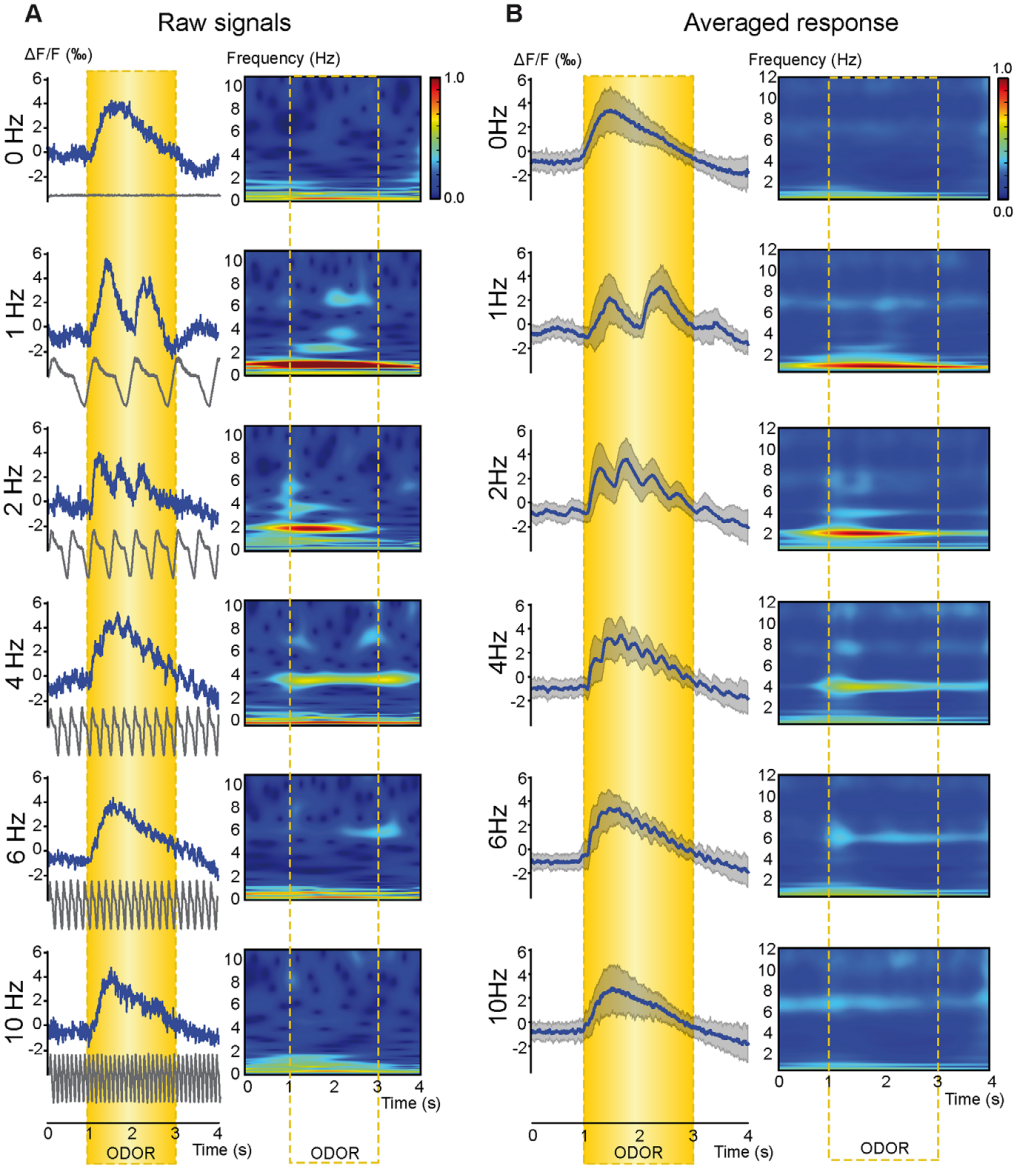
Impact of sniffing frequency on VSDi signal dynamics. A: An example of a raw VSDi signal time-course (left) computed from the activated areas (see Fig. 1 and material and methods) and corresponding time-frequency representations (right). The black traces at the bottom of each panel represent the imposed sniffing signal recorded at the nostril entrance. Each line corresponds with a different sniffing frequency. Responses were recorded using a 1000 ml/min flow rate. The color scale (arbitrary unit) was the same for all time-frequency representations. B: Averaged optical responses (blue line) ± SD (shadow area) computed from all animals according to the different sniffing frequencies (left), regardless of flow rate (0 Hz, n = 59; 1 Hz, n = 65; 2 Hz, n = 151; 4 Hz, n = 123; 6 Hz, n = 75; 10 Hz, n = 63). The corresponding mean time-frequency representation (right) computed from all individual time-frequency representations. The color scale (arbitrary unit) was the same for all time-frequency representations. Yellow box indicates odor stimulation period.

Previous studies using Ca^2+^ imaging of receptor cell terminals in the OB showed that the phasic response component decreased with increased sniffing frequency and even disappeared at a frequency of around 6 Hz [22], [24], [25]. Here, we used several sniffing frequencies (up to 10 Hz) within the physiological range. In the raw glomerular responses, a phasic component could be observed at frequencies up to 4 Hz. This sampling-related modulation decreased with increasing frequencies but was still observable at 6 Hz, as shown in the time-frequency representation (Fig. 2A). The phasic component of the glomerular optical response disappeared at the highest imposed sniffing frequency (10 Hz). Even if individual examples of raw VSDi data demonstrated an impact of sniffing on signal dynamics, one could raise the question of reproducibility in such observations. Because the respiratory dynamics were imposed by the sniffing simulator, the experimental protocol timing was highly reproducible across recordings for a given frequency. Using this reproducibility, we averaged the VSDi responses from all of the responses evoked by a given sniffing frequency, regardless of the flow rate and odor used. As shown in figure 2B, sampling-related modulation was clearly distinguishable from the mean signal with a sampling frequency up to 6 Hz, which demonstrates that the modulation observed in one rat (Fig. 2A) was a general feature of the VSDi glomerular responses. It should also be noted that high-frequency sniffing (see 4 Hz or 6 Hz in figure 2B) led to a stepwise rising phase before reaching the maximum level. To confirm these results, we generated a mean time-frequency representation: time-frequencies representations were computed from all individual responses and then averaged across the responses for each frequency. Except for 10 Hz, the mean time-frequency representations exhibited an increased power during odor presentation in the frequency band corresponding with the imposed sniffing frequency (Fig. 2B). This result was used to quantify the sniffing modulation and demonstrate that the power increase observed in the time-frequency representation was not only a frequency component or harmonic of the tonic response. We extracted the maximum power of the time-frequency representation in each frequency band of interest and compared it with the frequency content of continuous simulation (0 Hz), which only evoked a tonic component in the response, using a one-way ANOVA. The power increase in the optical responses corresponding with the imposed sniffing frequency was significantly higher than the corresponding frequency content of the tonic component for 1 Hz (F_(1,122)_ = 78.2, P<0.0001), 2 Hz (F_(1,208)_ = 143.3, P<0.0001), 4 Hz (F_(1,180)_ = 97.2, P<0.0001), and 6 Hz (F_(1,132)_ = 35, P<0.0001) but not 10 Hz (F_(1,120)_ = 1.47, P = 0.23) (Fig. S3A).

**Figure 3:**
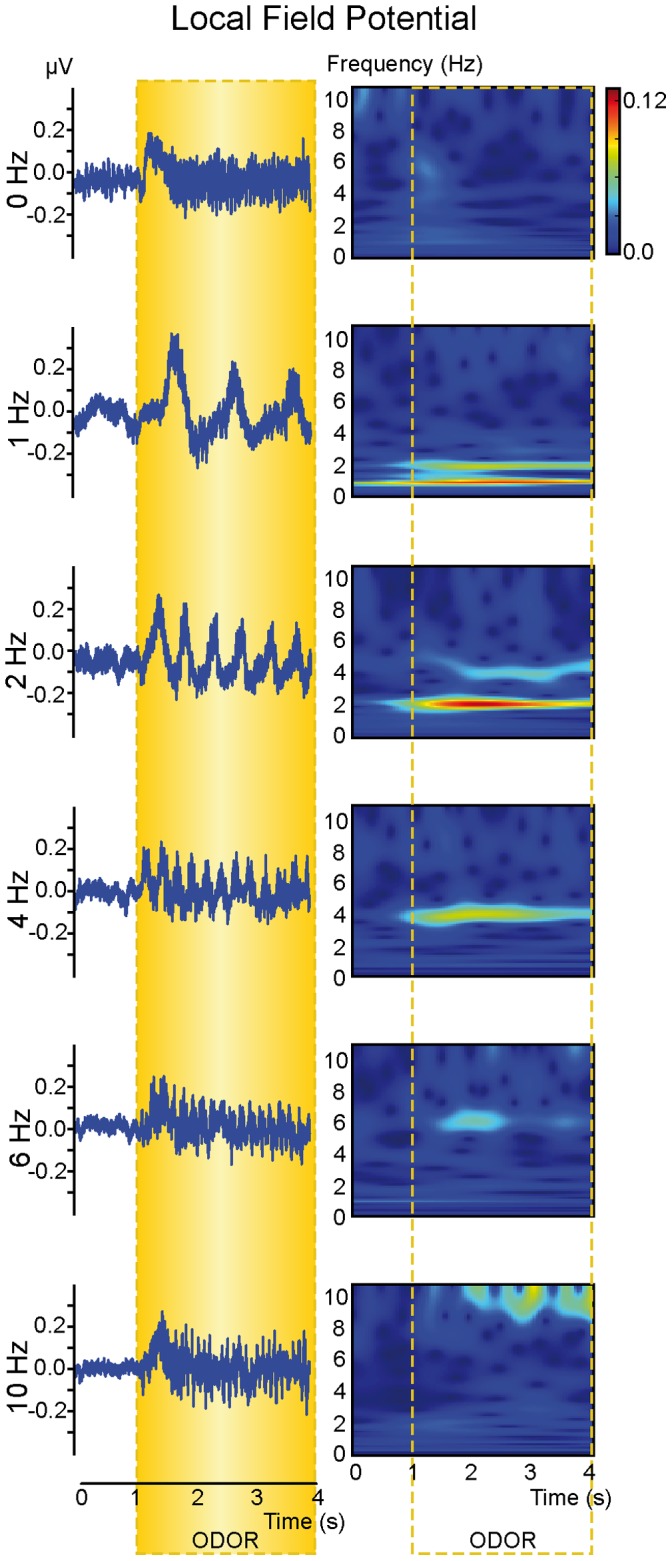
Impact of sniffing frequency on LFP signals. An example of local field potentials recorded from the granule cell layer (left) and corresponding time-frequency representations (right). The responses were recorded using a 1000 ml/min flow rate. The color scale (arbitrary unit) was the same for all time-frequency representations. Yellow box indicates odor stimulation period.

We also wanted to verify that the lack of a phasic component at 10 Hz was not related to a limitation of our artificial sniffing set-up. First, a 10-Hz modulation was observed for some optical responses (Fig. S4), even if such modulation was not statistically present when averaging responses. Second, we applied the same experimental paradigm for the LFP recordings. As shown in figure 3, LFPs also developed a phasic response locked to the imposed sampling. As observed in the VSDi responses, the LFP sniffing modulation amplitude decreased with increasing frequency even if the decrease was weak compared with the optical response. Interestingly, LFP signals remained clearly modulated by sniffing at the highest imposed frequency (10 Hz). This result was confirmed by a quantitative analysis based on time-frequency representation (Fig. S3B). These data also confirmed that although a 10 Hz modulation was not always present in the optical response, the OB network was still able to exhibit sniffing modulation.

**Figure 4:**
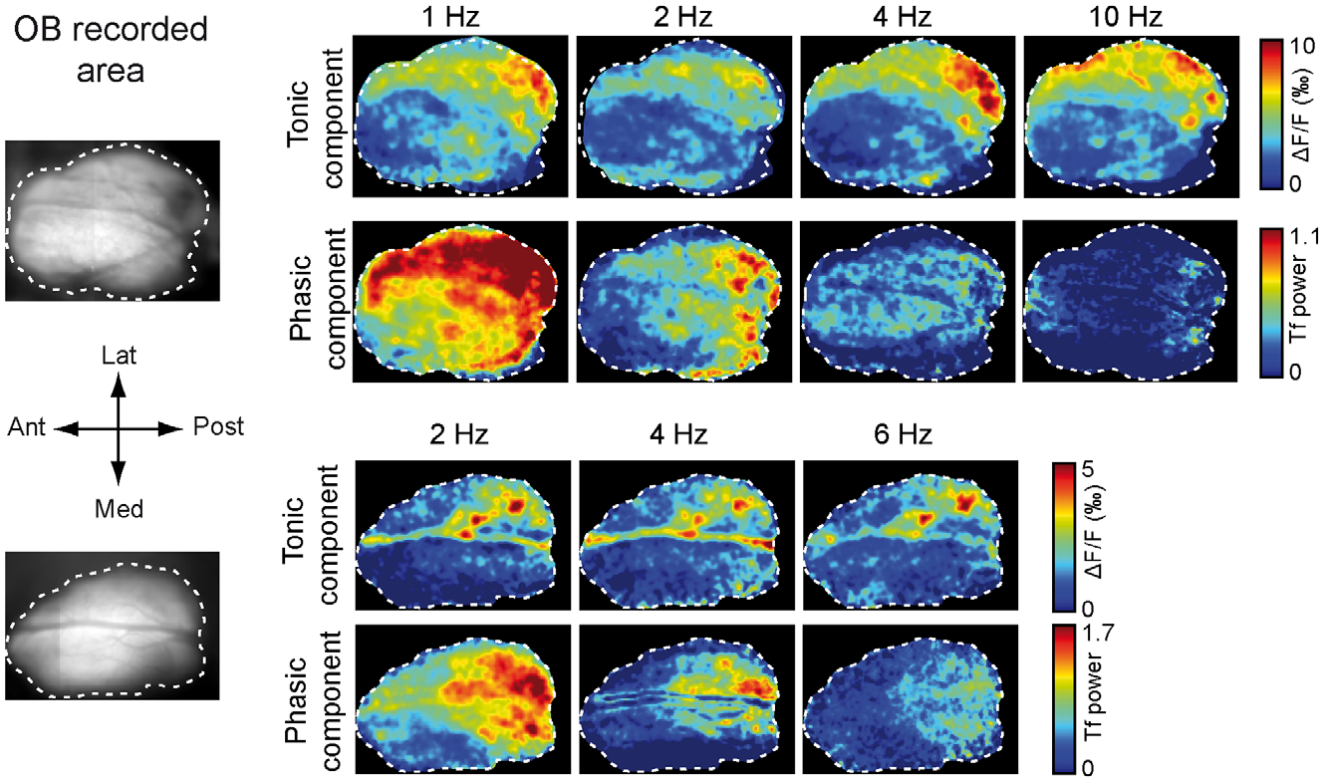
Spatial distribution of the tonic and phasic component amplitudes as a function of sniffing frequency. Two examples of odor-evoked maps computed from two different rats as a function of sniffing frequency. The first column shows the recorded area of the dorsal OB. In each panel, the first row displays the spatial distribution of the odor-evoked activity computed from the early tonic responses (see material and methods), i.e., the early-responding glomeruli. Color scale (arbitrary unit) was the same for all maps in a row. Due to dye bleaching, signal to noise ratio sometimes decreased along the experiment leading to difference in signal amplitude between different maps. The second row displays the spatial distribution of the phasic component amplitude. We performed a pixel-by-pixel time-frequency analysis, and for each pixel, we extracted the maximum power of the time-frequency map in the frequency band of interest. Color scale (arbitrary unit) was the same for all maps in a row.

Lastly, we wanted to identify how sniffing modulation could impact the spatial distribution of odor-evoked activity. The data shown in figure 4 shows that sniffing frequency had little effect on the activation map computed from the tonic response. Indeed, the spatial distribution of early-responding glomeruli remained similar, independent of the imposed sniffing frequency. We also performed the first spatial distribution analysis of the phasic VSDi response component by developing a pixel-by-pixel time-frequency analysis. We observed that the phasic component power map largely overlapped with the tonic component map (Fig. 4), which indicated that sniffing modulation was spatially concomitant with the odor-evoked response. Nevertheless, there was an obvious decreasing in the spread of the phasic component when the sampling frequency was increased.

### Effect of Frequency on the Tonic and Phasic Components of the VSDi Signal Amplitude Depends on Flow Rate

When exploring their environment, rats varied both their sniffing frequency and flow rate [21]. After having demonstrated that OB activity could be maintained at high-frequency sniffing, we investigated how the sniffing flow rate impacted OB activity concomitantly with sniffing frequency.

#### Tonic response

**Figure 5:**
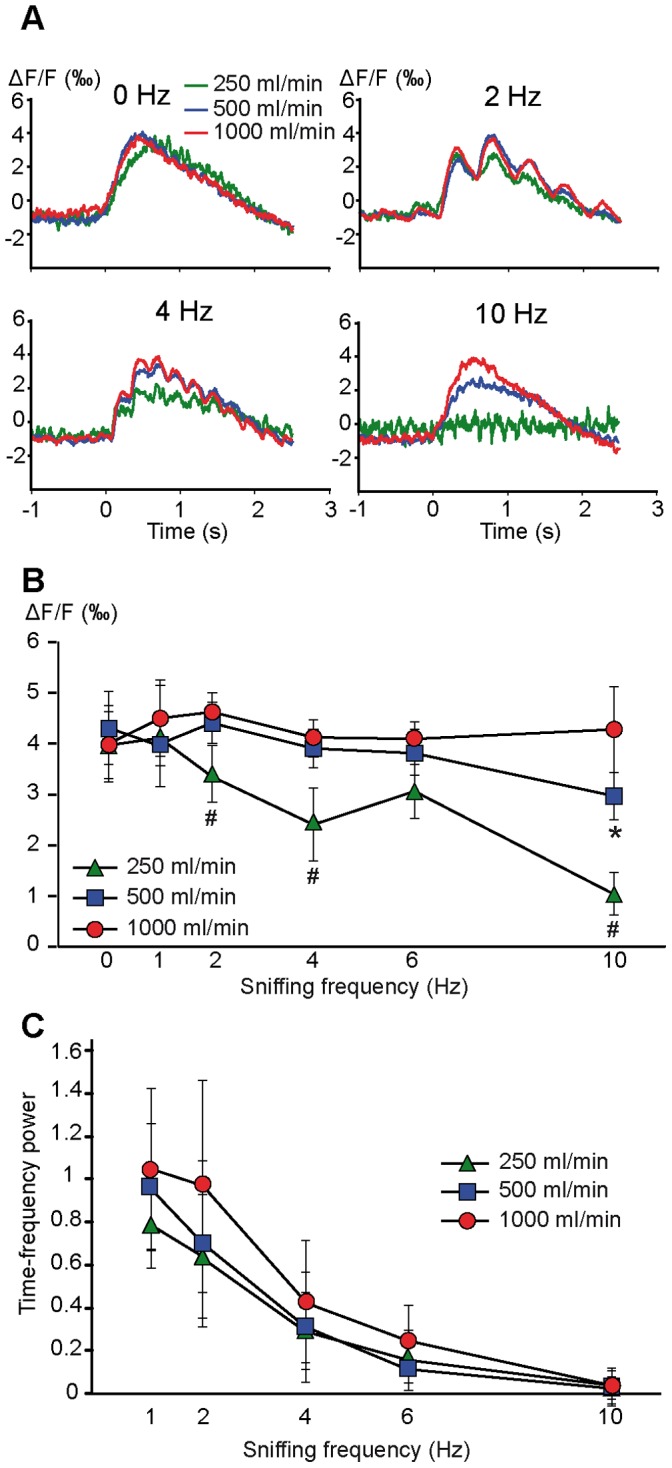
Concomitant effect of sniffing frequency and flow rate. A: Mean optical response as a function of sniffing frequency and flow rate. B: Mean amplitude (±SD) of the tonic component of the optical responses as a function of both flow rate and frequency. (*) and (#) indicate that the amplitude of the tonic response was significantly different for 500 ml/min and 250 ml/min, respectively. C: Mean amplitude (±SD) of the sampling-related modulation (phasic component amplitude) as a function of both sniffing frequency and flow rate.

The mean VSDi signals computed as a function of sniffing frequency regardless of flow rate indicated that sniffing frequency had little impact on the amplitude of the VSDi tonic component responses (Fig. 2B). To evaluate the extent to which the flow rate impacted VSDi responses concomitantly with frequency, we performed a quantitative analysis of the VSDi tonic component amplitude. All descriptive statistics are detailed in supplementary Table S1. As shown in figure 5A, flow rate had little impact on optical responses in low-frequency sniffing (2 Hz). Conversely, the flow rate appeared to impact the response amplitude at high sniffing frequencies. A two-way ANOVA on signal amplitude with flow rate and sniffing frequency as factors confirmed significant effects of both flow rate (F_(2, 518)_ = 19.8, P<0.0001) and frequency (F_(5, 518)_ = 8.95, P<0.0001) and a significant interaction between these two factors (F_(10, 518)_, = 2.756, P<0.01). Partial ANOVAs for each flow rate confirmed that sniffing frequency had no significant effect on signal amplitude at a high flow rate (1000 ml/min, F_(5, 242)_ = 1.16, P>0.1; see figure 2A). Conversely, for moderate (500 ml/min) and low (250 ml/min) flow rates, there was a statistically significant effect of increased sniffing frequency on the tonic component amplitude (F_(5, 205)_ = 4.62, P<0.001 and F_(5, 71)_ = 11.7, P<0.0001, respectively). Post-hoc analyses of these data confirmed a decrease in tonic component amplitude only for the highest sniffing frequency (10 Hz) when using a moderate flow rate and an earlier frequency increase effect when using a low flow rate (Fig. 5B).

#### Phasic response

We next investigated the impact of both sampling parameters on the phasic sampling-related component of the optical responses (Fig. 5C). Both sampling frequency (F_(4, 462)_ = 93.714, P<0.0001) and flow rate (F_(2, 462)_ = 8.642, P<0.001) had a significant impact on the phasic component amplitude of the response (see all descriptive statistics in supplementary Table S2). The phasic component amplitude decreased with increasing frequency and decreasing flow rate. We did not observe a significant interaction between flow rate and frequency. In contrast to its effect on the tonic response, flow rate only partially counterbalanced the frequency effects.

## Discussion

Sniffing behavior is one of the key features of mammal olfactory perception, as sensory input is intimately related to respiration. Behavioral studies have already highlighted the importance of sniffing modulation in both flow rate and frequency during odor discrimination and detection tasks [21] or after novel odor presentation [33], [34]. The present work was the first attempt to analyze the concomitant effects of both frequency and flow rate on odor-evoked glomerular maps using VSDi. Using our newly developed artificial sniffing simulator, which allowed us to reproduce rat sniffing behavior at frequencies of up to 10 Hz, our data demonstrated that sniffing frequency and flow rate jointly impact the level and the dynamic of glomerular responses. These results bridged the gap between previous data obtained by Oka et al. [23] at the OB input level, and data from Courtiol et al. [13], [27] at the OB output level. Indeed, if Oka et al. [23] showed main impact of sniffing flow rate on the response amplitude, they only analyzed effects of sniffing frequency and flow rate separately. Moreover, the low temporal resolution of their recording method did not allow them to analyze the dynamics of sniffing related modulation of optical responses. Combined with the first time-frequency analysis of optical signals, VSDi showed that high sniffing frequency retained the ability to modulate OB activity at the glomerular level.

### Respiration Related Modulation can be Maintained in High-frequency Sniffing

Contrary to previous studies showing that sniffing modulations of odor-evoked glomerular optical responses tend to disappear at frequencies around 5–6 Hz [22], [24], [25], our results showed that odor-evoked OB activity remained modulated in high-frequency sniffing both at the glomerular and intrinsic network levels. This difference can be first explained by our imaging approach. Indeed, all previous studies focusing on the impact of sniffing frequency were based on Ca2+ imaging of presynaptic activity. Voltage-sensitive dyes are primarily used to visualize post-synaptic activity [28], and we could not exclude the hypothesis that input activity could be reshaped by local circuits at the glomerular level, thus maintaining sniffing modulation at the post-synaptic level. External tufted cells are entrained by repetitive sensory input at frequencies of up to 10 Hz [35]. Ensembles of entrained tufted cells of a given glomerulus may synchronize glomerular network activity and amplify rhythmic activity at the sniffing frequency [35], [36]. This hypothesis agrees with the LFP data showing that intrinsic OB network activity was still modulated by sniffing at 10 Hz and was confirmed by a recent study showing that mitral cells maintain their sampling-related modulation in high-frequency sniffing [27]. Other methodological issues could be taken into account, such as odor choices [22], [24] or the use of a sniffing simulator which reproduce both respiratory phases unlike previously used devices [23], [24]. In our anesthetized model, only bottom-up processes could influence OB activity, and we could not rule out that top-down control may explain differences observed with awake model [22], [25]. Finally, our quantification analysis revealed sniffing frequency effects that could not be observed by a simple visual observation. Our data confirmed that the sniffing modulation of optical signals decreased with increasing frequency [22], [24], [25] independent of the imposed flow rate. This decrease could explain the difficulty in observing the sniffing-related modulation of optical signals at 10 Hz, which could be related to a low signal-to-noise ratio. Indeed, rats exhibiting the largest response amplitude also displayed sniffing modulation at 10 Hz. This result and the presence of a phasic component at 10 Hz on LFP signals indicate that the lack of sniffing modulation was not due to temporal filtering by VSDi or a limitation of our artificial sniffing setup.

What is the importance of the persistence of sampling-related dynamics? It has been shown that sniffing behavior is coupled with head movements and whisking, which are also involved in exploratory behavior [37], [38]. These rhythms appear in the same frequency band as the hippocampal theta rhythm. Sniffing and theta rhythms not only share similar frequencies but also exhibit phase relationships [39]. Moreover, OB sniffing-related rhythms and/or hippocampal theta rhythms appear to be synchronized during odor sampling [40]. Thus, sniffing or theta rhythms could allow coupling between olfactory structures and other brain regions, and the persistence of sniffing-related activity at high sniffing frequencies could be a key mechanism in communication between brain areas [19].

### Tonic and Phasic Components of VSDi Responses are Differentially Affected by Sampling Parameters: do Tonic and Phasic Components Mirror Flow Rate and Inspiration Volume, Respectively?

At high flow rates, the tonic component amplitude of the VSDi responses was independent of sniffing frequency. This result agrees with previous data showing that olfactory nerve terminal response amplitude was only slightly affected by sniffing frequency [23]. These data also agree with results from Cury and Uchida [41] showing that changes in sniffing frequency weakly impact mitral cell discriminability. A major impact of flow rate on OB activity has also been reported in both the mitral cell discharge and LFP oscillation levels [27]. Nevertheless, even if the tonic component amplitude was not affected by sniffing frequency at a high flow rate, increasing frequency leads to a stepwise rising phase before reaching the maximum level, which indicates that maximum receptor activation likely requires cumulative activation across successive respiratory cycles. The effect of sniffing frequency appeared at moderate and low flow rates, which indicates that the primary impact on the post-synaptic glomerular tonic response is a factor of flow rate rather than frequency. Such a concomitant impact of sniffing flow rate and frequency was not reported by Oka et al. [23], but they only analyzed effect of sniffing frequency at moderate flow rate. In our study, a decrease in tonic component amplitude was only observed for the highest sniffing frequency (10 Hz) when using a moderate flow rate, and the main impact of sniffing frequency was reported for the low flow rate. Our data also showed that the spatial distribution of the tonic component amplitude was only slightly modified by an increase in sniffing frequency using a high flow rate, suggesting that odor spatial representation at the OB input level is stable across the physiological range of sniffing frequencies. Conversely, sniffing frequency highly impacted the phasic response amplitude. Regardless of the flow rate, the phasic component amplitude significantly decreased with increasing frequency. The relationship between the phasic component amplitude and sniffing frequency (Fig. 5C) was similar to the relationship between inspiration volume and sniffing frequency (Fig. S5), which suggests that the sniffing-related modulation of VSDi responses largely depends on the volume of each inspiration. The phasic component was also affected to a lesser extent by flow rate. Even if a decrease in phasic component amplitude with increasing sampling frequency was a general feature of optical responses, a high flow rate helped maintain a higher level of sampling-related modulation.

Taken together, our results suggest that both sniffing parameters jointly affect odor-evoked responses. If increased sniffing frequency could affect the odor-evoked tonic response at moderate and low flow rates, a decrease could be compensated by an increased flow rate; for example, we found that at 10 Hz, an increased flow rate of 1000 ml/min restored the signal amplitude observed at 2 Hz. This synergic effect of flow rate and frequency is in accordance with electrophysiological and behavioral data [27], [42].

### Conclusions

Using an artificial sniffing paradigm in anesthetized rats, our main goal was to analyze bottom-up influence of sniffing behavior on odor representation at the OB input level. Our data demonstrated that sniffing frequency and flow rate jointly impact the level and the dynamic of glomerular responses. Whereas high sniffing frequency retained the ability to shape OB activity, increasing sniffing frequency has little impact on glomerular response amplitude when combined with a high flow rate. In other words, the post-synaptic representation of odors at the glomerular level could be maintained in high-frequency sniffing if the increase in frequency is compensated by an increase in flow rate. Such a co-variation of both parameters has been observed in behaving rats [21], [42]. Thus an animal has the ability to combine various sniffing parameters differently according to the odor, task and/or context. If top-down processes may also influence OB activity in awake animals, such sensory mechanism provides a stabilized odor representation independent of the sniffing behavior adopted by the animal during odor detection or recognition and may help maintain an intensity invariance of the odor representation at the OB input level [18]. As suggested by Schoenfeld and Cleland [43], [44], adaptive sampling may also improve olfactory capabilities by allowing the optimization of the deposition of odor molecules through the olfactory epithelium. Such hypothesis agrees with data from Oka et al. [23] showing that flow rate effect depends on the odorant chemical properties. However, if frequency does not alter odor representation, what is the functional role of sniffing behavior? Increased sniffing frequency was not observed in head-restrained models except for the first presentation of a new odorant [25], [34]. Increased sniffing frequency was observed in freely moving rats in which the odor is actively delivered to the animal at a precise localization [19], [45]. Nevertheless, odor could be identified in one sniff indicating that prolonged high frequency sniffing seems not necessary for odor discrimination in such protocol. A clear modulation of sniffing parameters has been observed in animals that actively sample their environment [21]. Thus, this behavior could be driven by expectation or movement toward an odor source to spatially localize the odor. A fast-sniffing mode could facilitate odor acquisition through the odor plume when tracking an odor source, allowing the rapid detection of fine changes in odor concentration. Rapid sniffing could also allow suppression of background signals, thus helping detect new odorants [25]. Finally, the rapid repetitive sampling of inputs could allow the bulbo-cortical network to construct perceptual hierarchies to use in recognizing environmental odor cues [46].

## Materials and Methods

### Ethics Statement

Animal protocols were conducted in agreement with the French (council directive 87848, October 19, 1987) and international (directive 86-609, November 24, 1986, European Community) legislation for animal experiments, and received approval from the Lyon 1 University Ethics Committee (permission #69387473). Care was taken at all stages to minimize stress and discomfort to the animals.

### Subjects

Male Wistar rats (Centre d’Elevage et de Recherche Janvier, Le Genest-St-Isle, France) weighing 220–260 g were housed in standard rat cages (polycarbonate, 49×26×20 cm) in a temperature-controlled vivarium with a 12 h reverse light cycle (light on at 8:00 P.M.). All rats were given access to food and water.

### Surgery

Animals were anesthetized using urethane (1.5 g/kg i.p., with additional supplements as needed). Body temperature was maintained between 36.5°C and 37.5°C using a heating pad and rectal probe. To control sniffing parameters, the trachea was incised and cannulated with two tubes, a tracheal cannula leading to the lung (Catheter Biotrol, internal diameter 1.57 mm, external diameter 2.08 mm) and nasal cannula leading to the postnasal cavity through the pharynx (catheter Vygon, Venolux 247, internal diameter 0.8 mm). After double cannulation, rats were fixed on a stereotaxic frame, and using a dental drill, the skull overlying the dorsal surface of the OB was thinned and then removed. The dura matter was carefully removed using a tungsten wire.

### Dye Staining

A plastic chamber (external diameter 8 mm, internal diameter 5 mm, height 1 mm) was fixed on the skull using dental cement (Unifast Trad), and was then filled with dye solution. The dye used was RH-1838 (Optical Imaging Inc.), which is a blue oxonol dye shown to work well in the rat OB [28]. This dye molecule transforms changes in membrane potential into optical signals that occur in microseconds allowing to record neural activity with millisecond precision [47], [48]. The dye was dissolved in artificial cerebrospinal fluid (aCSF, Phymep) until the dye-containing solution had an optical density of 4–7 measured at 580 nm. The OB was stained for 2–2.5 hr. At the end of the staining, the chamber and olfactory bulb were rinsed, and the chamber was filled with 1.5% agarose and enclosed with a cover glass.

### Optical Imaging

The olfactory bulb surface was illuminated using an epi-illumination system with a 630 nm interference filter for excitation (bandwidth of 30 nm), a dichroic mirror (650 DLRP), and a 665 nm long-pass filter for emission. Images were acquired using a commercial brain imager (imager 3001/M, Optical Imaging Inc.) mounted on a tandem photo lens system (Nikkor 50 mm, f = 1.2 and Nikkor 135 mm, f = 2), leading to a 2.7x magnification. Images were spatially binned 4×4 (final pixel size 20×20 µm) and digitized at 160 Hz. Each trial consisted of two consecutive blocks of images (5 sec) triggered on the rat’s tracheal respiration, the C0 control block and the C1 test block. During C0, neither airflow nor odorant stimulation was applied through the nasal cavity during OB imaging. During C1, the odor was applied through an artificial sniff as described below. For each condition, five trials were averaged to increase the signal-to-noise ratio.

### Electrophysiological Recordings

Bulbar activity was recorded as a broadband signal (0.1 Hz to 5 kHz) using 16-channel silicon probes (NeuroNexus Technologies, Ann Arbor, MI) and a homemade 16-channel DC amplifier (gain 1000x). The 16-channel silicon probes were placed to ensure the ability to record the maximum LFP amplitude of the granular cell layer. The granular cell layer was located by the LFP waveform, as described by Buonviso et al. [12]. Recordings were performed in the whole antero-posterior axis of the OB. Data were digitally sampled at 20 kHz and acquired with a PC using a National Instrument acquisition card (BNC-2111).

### Odor Stimulation

Odors (isoamyl acetate (ISO), 2-heptanone (K07), hexanal (D06), and ethyl butyrate (BUT), Sigma Aldrich, Fluka) were delivered to the front of the animal’s nose using a custom-built flow dilution olfactometer with a concentration of ∼5.10-2 of saturated vapor and at a rate of 1 L/min. The recording protocol was as follows: 2 s of pre-stimulus recording, 2 s of olfactory stimulation recording and 1 s of post-stimulus recording. For LFP experiments, the recording period consisted of 2 s of pre-stimulus recording, 5 s of olfactory stimulation recording and 1 s of post-stimulus recording. The time delay between each odor presentation was at least 2 min.

### Imposed Nasal Airflow Protocol

To simulate respiratory cycles, we used a homemade apparatus that allowed the reproduction of both inhalation and exhalation phases [13]. To study the influence of airflow variation, we chose to impose three nasal airflow rates: low (250 ml/min), moderate (500 ml/min) and high (1000 ml/min). We also varied the sampling frequency by using values of 1, 2, 4, 6 or 10 Hz and a continuous nasal airflow (referred to as 0 Hz in the experiments) (Fig. S2). These flow rates and sampling frequencies corresponded with the physiological range of rats [21], [49]. The respiratory cycle simulator was triggered by the start of data acquisition.

Animal respiration was measured by a sensor placed in front of the tracheal cannula (Fig. S2). Actual airflow circulating through the nasal cavity was measured by a second sensor placed at the entry of the nostril. We used fast response time airflow sensors (bidirectional micro bridge mass airflow sensor, AWM 2000 series, Honeywell®). This setup has been previously described in detail [29].

### Data Processing

Data processing was performed using OpenElectrophy open-access homemade software [50]. OpenElectrophy is open source and freely available for download at http://neuralensemble.org/trac/OpenElectrophy. All signals and epochs (i.e., with or without odor) were stored in an SQL database.

#### Optical data processing

For each trial and pixel, the frame average over the first 200 ms was defined as the baseline fluorescence (F). This value was used to convert raw images to images that corresponded with the relative changes in fluorescence (ΔF/F). The optical signal was then detrended to eliminate fluorescence decrease due to bleaching.

Odor-evoked maps were computed after averaging five consecutive trials and low-pass filtering of the resulting signal (0–20 Hz). Maps were defined as the difference between the odor stimulation period and one-second pre-stimulus period. One to three different regions of interest (ROI) were defined for each odor. They corresponded to early-responding glomeruli (the first 50 ms after odor presentation) using a 2 Hz sniffing frequency and 1000 ml/min flow rate. These ROIs were used to compute the optical signal time-course for all other frequencies and flow rates. The amplitude of the tonic component (see Fig. 1) of each computed optical response was defined as the maximum fluorescence increase.

#### Analysis of the optical signal dynamics

Superimposed onto the tonic component, the VSDi signals also presented a phasic component linked to sniffing rate [28]. To preserve both time and frequency information, we used a time-frequency representation (TFR) based on a continuous wavelet transform. TFRs were constructed during the spontaneous and odor-evoked activities for each sniffing condition. Optical signals had no unit as they corresponded to relative changes in fluorescence (ΔF/F). In consequence, time-frequency power was expressed in arbitrary unit. To access the amplitude of the sniffing-related component, the maximum TFR power in each sniffing band of interest (i.e., 1, 2, 4, 6 and 10 Hz) was detected. This maximum was also detected in each sniffing band of interest in the 0 Hz (continuous flow rate) condition. This condition was used to evaluate the frequency content of the tonic component response. Then, the mean (±SEM) of the VSDi sniffing-related modulation was calculated and compared with the mean of the 0 Hz condition (e.g., the maximum value detected in the 2 Hz frequency band for the trial using a 2 Hz artificial sniffing frequency was compared with the maximum value detected in the same frequency band for the trial using a 0 Hz artificial sniffing frequency). This allowed us to determine the presence or absence of sniffing-related modulation.

The spatial distribution of the respiratory-related modulation was analyzed using a pixel-by-pixel time-frequency analysis. For each pixel, we extracted the maximum power of the time-frequency map in the frequency band of interest. For example, for a 2 Hz sniffing frequency, we computed the time-frequency representation at each point and plotted the maximum power at 2 Hz (±0.1 Hz). These values were used to represent the dorsal OB sniffing modulation map.

#### LFPs analysis

LFPs were analyzed after band-passing the signal at 0–200 Hz. Then, the TFR of the LFP signals were computed to visualize the sniffing-related modulation amplitude. The TFR was computed using the same parameters as the optical responses, and the presence of a phasic component was evaluated by comparing the sampling-related modulation amplitude in each frequency band of interest with the frequency content of the 0-Hz-evoked response (see above).

### Statistical Analyses

Data from 20 rats were included in data analysis for a total of 536 recordings. To analyze the maximum signal variation, a two-way analysis of variance (ANOVA) was used with flow rate (250 ml/min vs. 500 ml/min vs. 1000 ml/min) and frequency (0 Hz vs. 1 Hz vs. 2 Hz vs. 4 Hz vs. 6 Hz vs. 10 Hz) as factors. Partial ANOVAs and Student-Newman-Keuls post hoc tests complemented the analysis when appropriate. The threshold for rejecting the null hypothesis was 0.05. For sniffing-related modulations, each maximum value detected in the frequency band of interest was compared with the maximum value detected in the same band for the 0 Hz condition using a one-way ANOVA with the sniffing frequency (0 Hz vs. 1 Hz; 0 Hz vs. 2 Hz; 0 Hz vs. 4 Hz; 0 Hz vs. 6 Hz; and 0 Hz vs. 10 Hz) as factor.

## Supporting Information

Figure S1 **Nasal airflow measurements.** Examples of imposed airflow recorded at the nostril entrance (see figure S2) at different frequencies and flow rates. The actual sniffing frequency through the nostril perfectly fitted with imposed sampling, and the actual flow rate remained proportional to the imposed flow rate across the entire frequency range.
(TIF)

Figure S2 **Description of the experimental paradigm.** Airflow through the nose was imposed via the nasal cannula allowing to mimic rat sniffing behavior. As in previous experiments in freely breathing rats [12], [15], odor was delivered in front of the nostril via a custom-built olfactometer. Odor sampling was imposed by nasal airflow. Airflow circulating through the nasal cavity was measured by a sensor placed at the entry of the nostril. Such device did not allow to record absolute flow rate because airtightness around the nostril was not total. Nevertheless, airflow measurement was directly proportional to absolute airflow [29].
(TIF)

Figure S3. **Amplitude of the sampling-related modulation.** A: Distribution of the averaged amplitude (±SD) (gray bars) of sniffing related modulation for VSDi signals according to sniffing frequency. Averaged values were computed from all raw time-frequency representations from which the maximum power in each frequency band of interest was extracted. The open bars correspond to the frequency content in each frequency band of the tonic response evoked by continuous flow (0 Hz, n = 59) in the nasal cavity. The phasic component amplitude of optical responses was significantly larger than the frequency content of the tonic component for 1 Hz (n = 65), 2 Hz (n = 151), 4 Hz (n = 123), and 6 Hz (n = 75) but not 10 Hz (n = 63) (see main text). B: Similar results were obtained for the LFP recordings, excluding the 10 Hz imposed frequency, which remained able to induce a phasic component significantly higher than the 10 Hz tonic component content (F_(1,72)_ = 41.1, P<0.0001).
(TIF)

Figure S4 **VSDi signal modulation at a 10 HZ sniffing frequency.** An example of a raw signal (left) that exhibited a sniffing related modulation at a 10 Hz sampling frequency. This modulation was confirmed by the time-frequency representation (right). Time-frequency power (arbitrary unit) is color-coded. It should be noted that this optical signal has a high signal-to-noise ratio (around 1%).
(TIF)

Figure S5 **Relationship between sampling frequency and inspiration volume.** The inspiration volume for each breathing cycle was directly proportional to the sampling frequency. Theoretical volumes were normalized relative to the inspiration volume at a 1 Hz sniffing frequency and 1000 ml/min flow rate.
(TIF)

Table S1 **Tonic component amplitude as a function of sniffing frequency and flow rate.** Mean ± SD of the tonic component amplitude computed as a function of sniffing frequency and flow rate. Number of signals in each group is indicated between brackets.
(DOC)

Table S2 **Phasic component amplitude as a function of sniffing frequency and flow rate.** Mean ± SD of the phasic component amplitude computed as a function of sniffing frequency and flow rate. Number of signals in each group is the same than in table S1.
(DOC)
