# Supplementary figures and images for: Faster, Deeper, Better: The Impact of Sniffing Modulation on Bulbar Olfactory Processing
Source: PLoS One. 2012 Jul 17;7(7):e40927. doi: 10.1371/journal.pone.0040927 (PMC3398873; doi:10.1371/journal.pone.0040927)

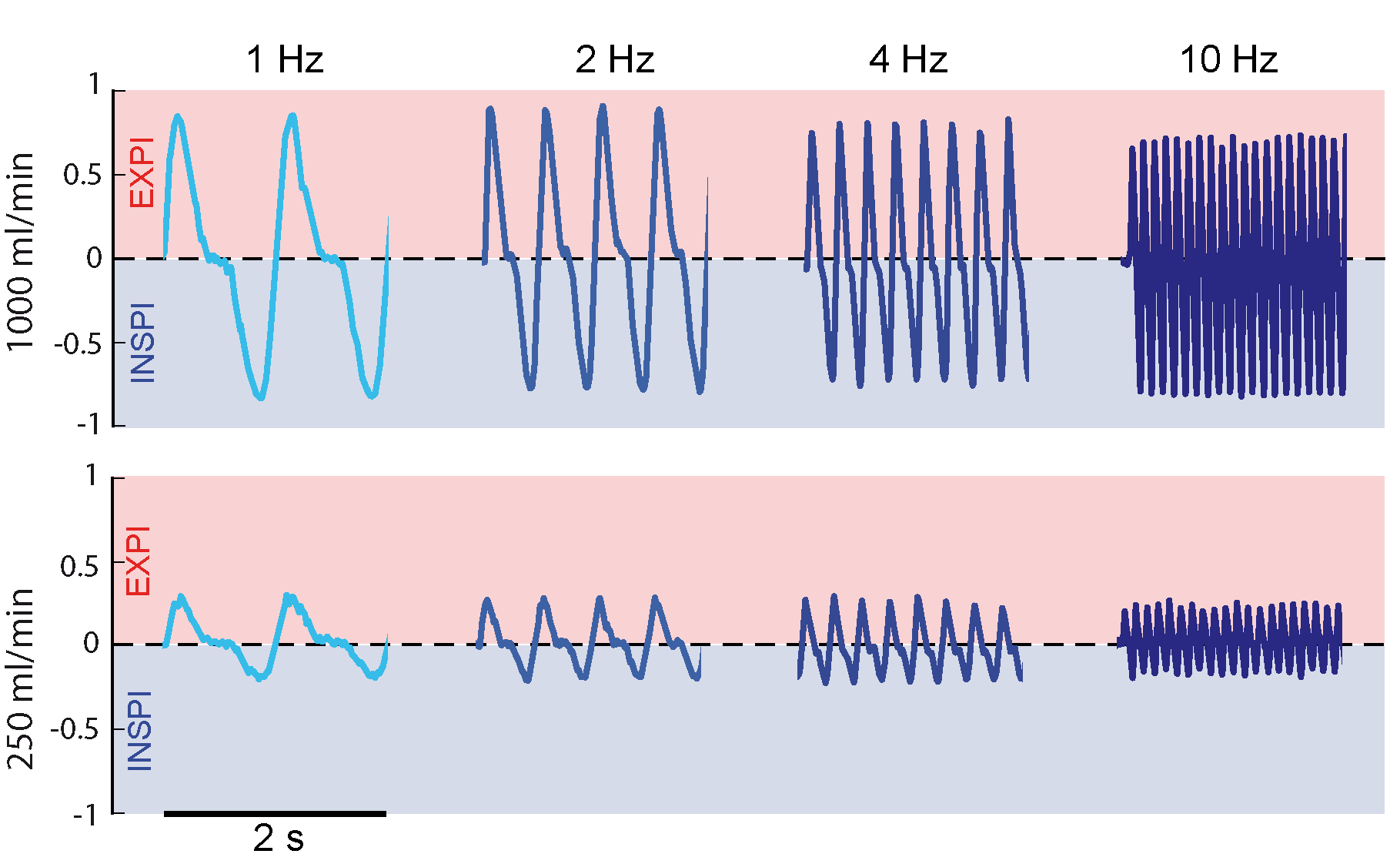

Supplement: Figure S1 — Nasal airflow measurements. Examples of imposed airflow recorded at the nostril entrance (see figure S2) at different frequencies and flow rates. The actual sniffing frequency through the nostril perfectly fitted with imposed sampling, and the actual flow rate remained proportional to the imposed flow rate across the entire frequency range. (TIF) [file pone.0040927.s001.tif]

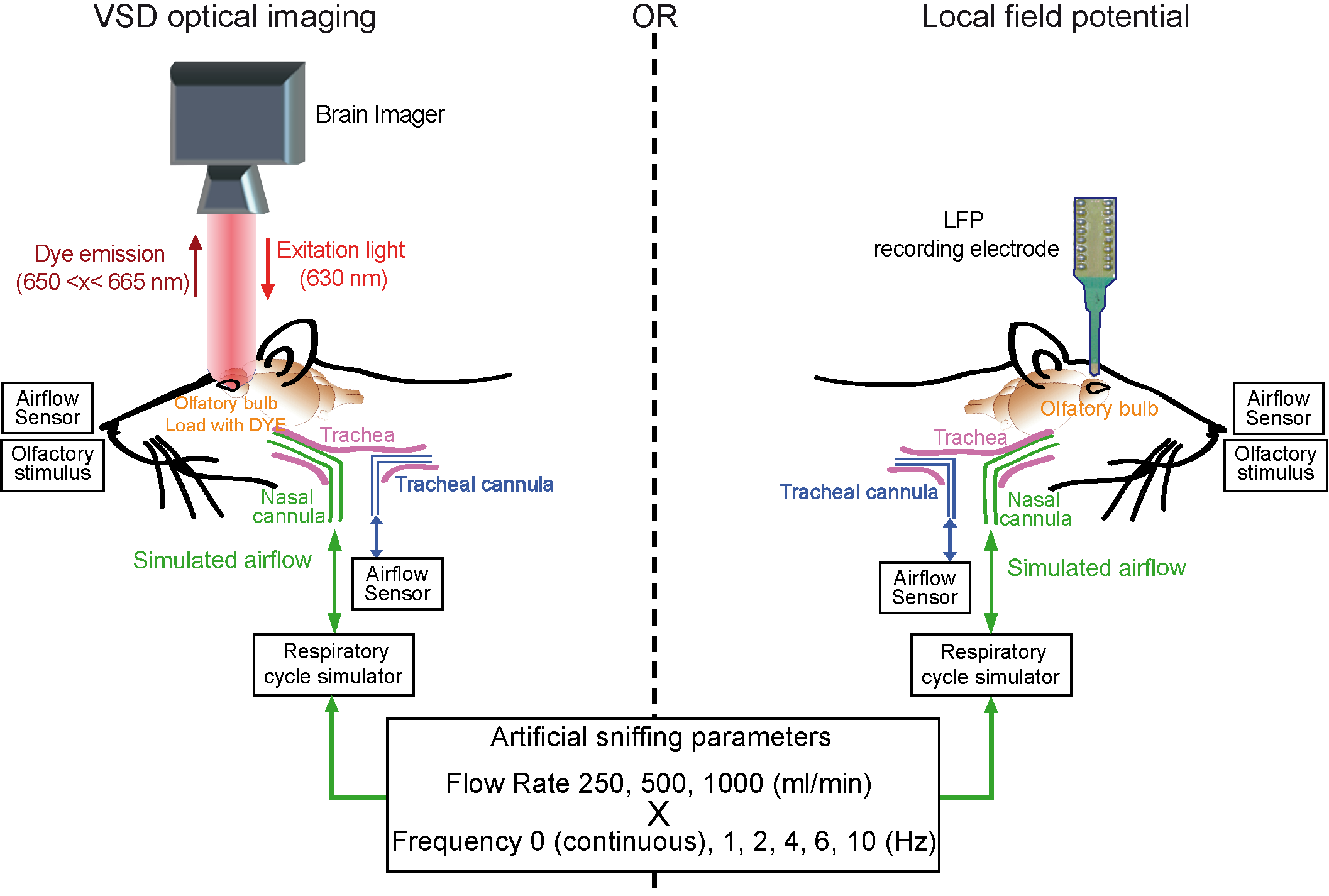

Supplement: Figure S2 — Description of the experimental paradigm. Airflow through the nose was imposed via the nasal cannula allowing to mimic rat sniffing behavior. As in previous experiments in freely breathing rats [12], [15], odor was delivered in front of the nostril via a custom-built olfactometer. Odor sampling was imposed by nasal airflow. Airflow circulating through the nasal cavity was measured by a sensor placed at the entry of the nostril. Such device did not allow to record absolute flow rate because airtightness around the nostril was not total. Nevertheless, airflow measurement was directly proportional to absolute airflow [29]. (TIF) [file pone.0040927.s002.tif]

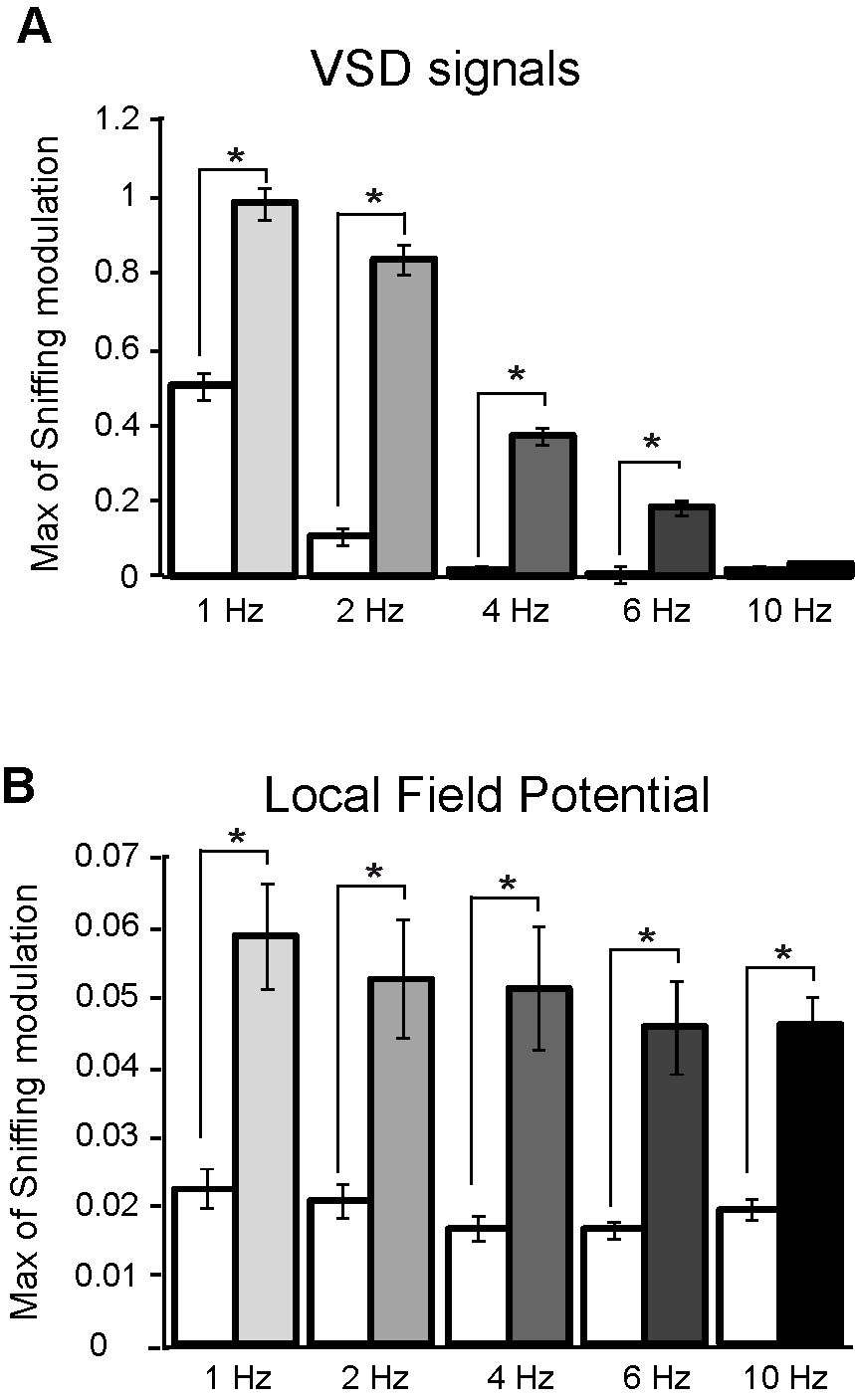

Supplement: Figure S3. — Amplitude of the sampling-related modulation. A: Distribution of the averaged amplitude (±SD) (gray bars) of sniffing related modulation for VSDi signals according to sniffing frequency. Averaged values were computed from all raw time-frequency representations from which the maximum power in each frequency band of interest was extracted. The open bars correspond to the frequency content in each frequency band of the tonic response evoked by continuous flow (0 Hz, n = 59) in the nasal cavity. The phasic component amplitude of optical responses was significantly larger than the frequency content of the tonic component for 1 Hz (n = 65), 2 Hz (n = 151), 4 Hz (n = 123), and 6 Hz (n = 75) but not 10 Hz (n = 63) (see main text). B: Similar results were obtained for the LFP recordings, excluding the 10 Hz imposed frequency, which remained able to induce a phasic component significantly higher than the 10 Hz tonic component content (F(1,72) = 41.1, P<0.0001). (TIF) [file pone.0040927.s003.tif]

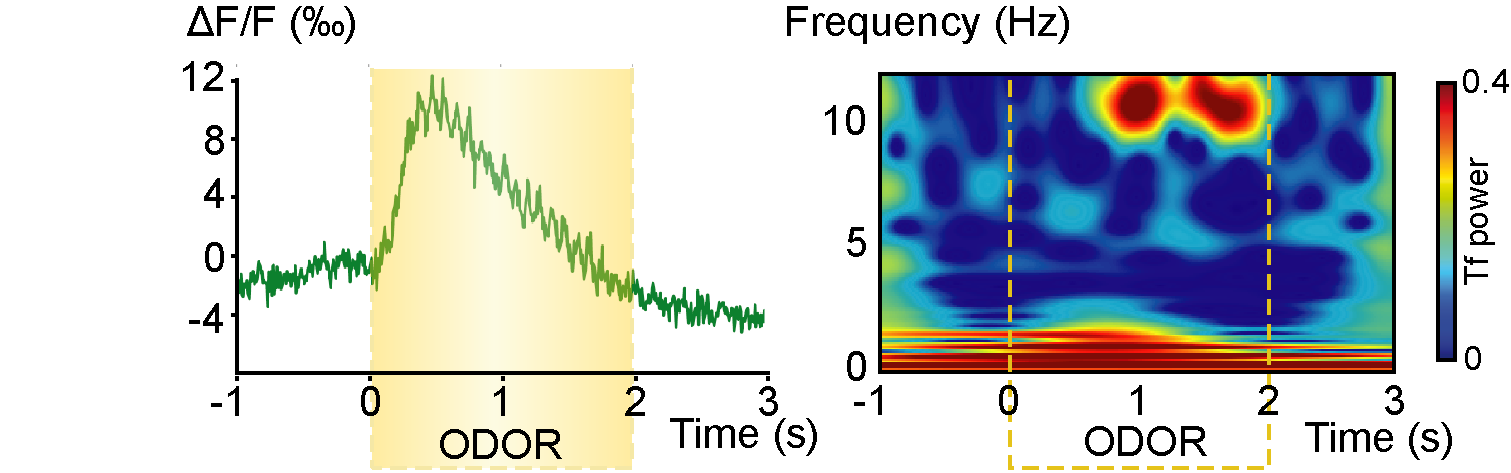

Supplement: Figure S4 — VSDi signal modulation at a 10 HZ sniffing frequency. An example of a raw signal (left) that exhibited a sniffing related modulation at a 10 Hz sampling frequency. This modulation was confirmed by the time-frequency representation (right). Time-frequency power (arbitrary unit) is color-coded. It should be noted that this optical signal has a high signal-to-noise ratio (around 1%). (TIF) [file pone.0040927.s004.tif]

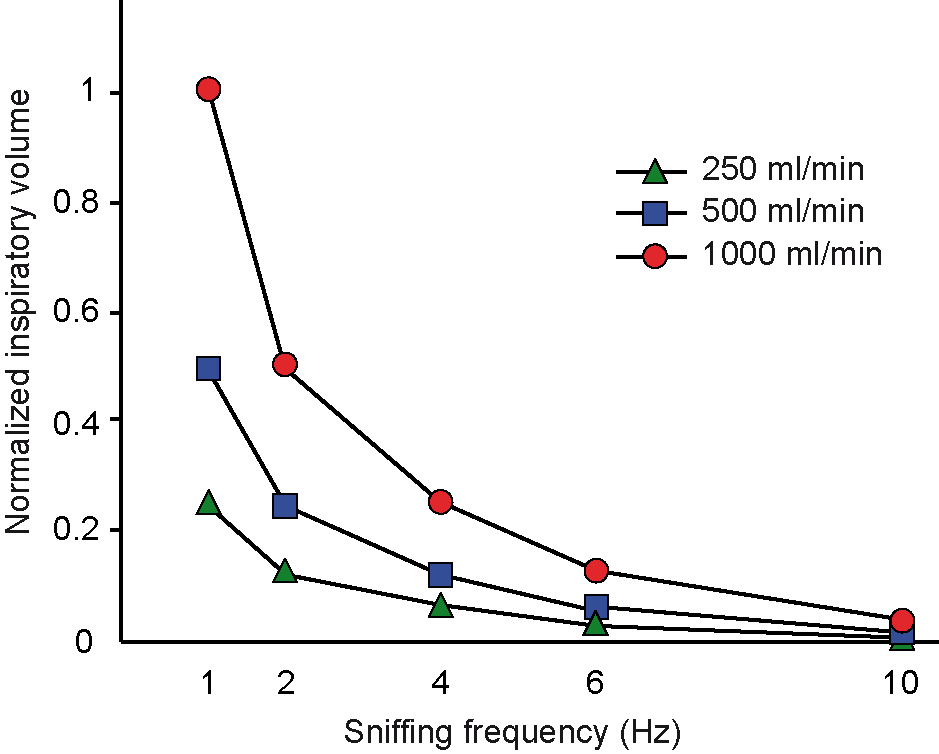

Supplement: Figure S5 — Relationship between sampling frequency and inspiration volume. The inspiration volume for each breathing cycle was directly proportional to the sampling frequency. Theoretical volumes were normalized relative to the inspiration volume at a 1 Hz sniffing frequency and 1000 ml/min flow rate. (TIF) [file pone.0040927.s005.tif]
