# Supplementary material for: Faster, Deeper, Better: The Impact of Sniffing Modulation on Bulbar Olfactory Processing
Source: PLoS One. 2012 Jul 17;7(7):e40927. doi: 10.1371/journal.pone.0040927 (PMC3398873; doi:10.1371/journal.pone.0040927)
Supplement: Table S1 — Tonic component amplitude as a function of sniffing frequency and flow rate. Mean ± SD of the tonic component amplitude computed as a function of sniffing frequency and flow rate. Number of signals in each group is indicated between brackets. (DOC) [file pone.0040927.s006.doc]

Table S1 : Tonic component amplitude as a function of sniffing frequency and flow rate

|  | 0 Hz | 1 Hz | 2 hz | 4 Hz | 6 Hz | 10 Hz |
| --- | --- | --- | --- | --- | --- | --- |
| 250 ml/min | 3.97 ± 1.07 (13) | 4.14 ± 1.19 (8) | 3.40 ±1.22 (20) | 1.4 ± 1.39 (17) | 3.05 ± 0.64 (8) | 1.05 ± 0.64 (11) |
| 500 ml/min | 4.3 ± 1.66 (23) | 4.00 ± 1.17 (31) | 4.40 ± 1.48 (54) | 3.92 ± 1.24 (45) | 3.82 ± 1.2 (30) | 2.97 ± 1.19 (28) |
| 1000 ml/min | 4.00 ± 1.75 (23) | 4.50 ± 1.82 (26) | 4.61 ± 1.65 (77) | 4.12 ± 1.4 (61) | 4.08 ± 1.09 (37) | 4.28 ± 1.98 (24) |

Mean ± SD of the tonic component amplitude computed as a function of sniffing frequency and flow rate. Number of signals in each group is indicated between brackets.
