# Supplementary material for: Faster, Deeper, Better: The Impact of Sniffing Modulation on Bulbar Olfactory Processing
Source: PLoS One. 2012 Jul 17;7(7):e40927. doi: 10.1371/journal.pone.0040927 (PMC3398873; doi:10.1371/journal.pone.0040927)
Supplement: Table S2 — Phasic component amplitude as a function of sniffing frequency and flow rate. Mean ± SD of the phasic component amplitude computed as a function of sniffing frequency and flow rate. Number of signals in each group is the same than in table S1. (DOC) [file pone.0040927.s007.doc]

Table S2 : Phasic component amplitude as a function of sniffing frequency and flow rate

|  | 1 Hz | 2 hz | 4 Hz | 6 Hz | 10 Hz |
| --- | --- | --- | --- | --- | --- |
| 250 ml/min | 0.78 ± 0.19 | 0.64 ± 0.28 | 0.29 ± 0.17 | 0.18 ± 0.12 | 0.03 ± 0.05 |
| 500 ml/min | 0.97 ± 0.29 | 0.70 ± 0.38 | 0.32 ± 0.25 | 0.12 ± 0.1 | 0.04 ± 0.08 |
| 1000 ml/min | 1.04 ± 0.37 | 0.97 ± 0.48 | 0.43 ± 0.28 | 0.23 ± 0.18 | 0.03 ± 0.08 |

Mean ± SD of the phasic component amplitude computed as a function of sniffing frequency and flow rate. Number of signals in each group is the same than in table S1.
